# The 5Cs of positive youth development: their impact on symptoms of depression, anxiety, stress, and emotional distress in Chilean adolescents

**DOI:** 10.1186/s40359-024-01863-x

**Published:** 2024-06-29

**Authors:** Mauricio Marín-Gutiérrez, Alejandra Caqueo-Urízar, Jenifer Castillo-Francino, Carolang Escobar-Soler

**Affiliations:** 1 Programa de Doctorado en Psicología, Universidad de Tarapacá, Arica, Chile; 2 Instituto de Alta Investigación, Universidad de Tarapacá, 18 de Septiembre 2222, Arica, Chile; 3 Centro de Justicia Educacional, Pontificia Universidad Católica de Chile, Santiago, Chile

**Keywords:** Positive development, Adolescents, Depression, Anxiety, Stress, Chile

## Abstract

Effective approaches to addressing mental health challenges faced by adolescents require a deep understanding of the factors contributing to optimal development, well-being, and prosperity. From the perspective of Positive Youth Development (PYD), this study proposes to examine the relationship between the 5Cs of PYD (Competence, Confidence, Connection, Character, and Caring) and symptoms of depression, anxiety, stress, and emotional distress among Chilean adolescents. A quantitative, cross-sectional, non-experimental study was conducted with 425 adolescents (ages 12 to 19, M = 14.95, SD = 1.81) from three Chilean cities: Arica (23%), Alto Hospicio (32%), and Iquique (46%). Data analysis included the use of confirmatory factor analysis (CFA) and structural equation modeling (SEM). The results indicate that two of the 5Cs, Confidence and Connection, have a significant negative direct effect on the four evaluated criterion indicators. These findings contribute to the literature on positive youth development in Latin America and underscore the importance of fostering confidence and connection in interventions aimed at promoting the mental health of adolescents in Chile and in similar contexts.

## Introduction

Adolescence is a crucial period of human development marked by significant physical, psychological, and social changes [1, 2]. Factors such as puberty, social pressure, and academic expectations converge to contribute to the complexity of the adolescent individual [3]. During this stage, it is common for teenagers to explore the boundaries of their world, often engaging in risky behaviors as they embark on the challenging task of consolidating their identity and constructing a life plan [4, 5].

The accumulation of stressful events and challenges inherent in their normative development can significantly impact their health, making them more prone to facing various mental health issues [6, 7]. Consequently, adolescents constitute a risk population for development of common mental disorders (CMDs), including depressive and anxiety disorders [8–10].

According to a recent meta-analysis [10], the overall prevalence of CMDs is estimated to range between 25% and 31% in adolescents. However, these figures must be considered alongside the significant increase in mental health issues during the COVID-19 pandemic lockdown period [11], where children and adolescents constituted the most affected vulnerable population by preventive measures [12].

These mental health issues are particularly concerning for society, not only due to their widespread prevalence in adolescents but also because of their long-term consequences and the burden they represent for public health [12]. Regarding their consequences, experiencing depression during adolescence can interfere with normal development, increasing the risk of facing a wide range of psychosocial problems in adulthood, including depressive and anxiety disorders, as well as alcohol and substance abuse or dependence, and a higher propensity for suicide [8, 13]. Similarly, experiencing anxiety carries similar consequences in adulthood due to the high comorbidity between depressive and anxiety disorders [14].

In terms of their burden on health, the 2019 Global Burden of Disease Study [15] highlighted that the two most disabling mental disorders worldwide were depressive and anxiety disorders. For the adolescent population in Chile, depressive and anxiety disorders rank among the top three causes of Disability-Adjusted Life Years (DALYs) for ages 10 to 14 years and 15 to 19 years [16], thus emphasizing the urgent need to address adolescent mental health in the country. Additionally, Quijada et al. [17] have shown that social inequality exacerbates mental health issues, with Chilean adolescents from lower socio-economic backgrounds facing increased risks due to factors such as poverty and limited access to resources.

This underscores a broader public health crisis, highlighting the critical need to prioritize access to mental health services for adolescents. Despite general awareness, a significant proportion of adolescents with mental health issues continue to lack access to these services, and when they do seek care, not all receive appropriate treatment [18]. In Chile, Vicente et al. [19] found that only one-third of children and adolescents with a psychiatric diagnosis received any mental health care, indicating a substantial treatment gap of 66.6%, which further increases to 85% when considering only the formal health system. More recently, Salinas-Contreras et al. [20] reported that only 16.5% of adolescents had ever used a mental health service for depression, with 9.7% receiving psychological treatment and 2.7% pharmacological treatment.

Given the evident crisis and the inadequacy in the provision of mental health services, the management of adolescent mental health in Chile requires a systemic approach that goes beyond mere clinical response. According to Martínez et al. [21], it is crucial to develop a comprehensive intervention strategy that not only combines treatment and prevention but also proactively and adaptively addresses the diverse needs of adolescents. This strategy must be flexible and evidence-based, allowing for the optimization of effective practices, adjustment of those that have not yielded results, and exploration of new approaches.

### The 5Cs of positive youth development (PYD) and their relationship with mental health

Effectively addressing the complexities and challenges in adolescent mental health requires a deep understanding not only of the risks factors but also of those protective factors that facilitate and promote healthy development [2]. In this context, the so-called Positive Youth Development (PYD) approach has gained relevance in social and health sciences by detailing the conditions and processes that contribute to optimal development, well-being, prosperity, and success among young people [22, 23].

The 5Cs model of PYD proposed by Lerner [1, 24], is as one of the most influential frameworks for understanding healthy adolescent development [25, 26]. It begins with the concept that young people are individuals with “resources to be developed” rather than incomplete, risky, or problematic beings [27]. In other words, all young people can thrive and embark on paths of healthy development when their personal strengths align with the support and opportunities provided by their family, peer group, school, and community [27–30].

From this understanding, positive development is manifested by the emergence of five internal characteristics, referred to as the “5Cs”, which enable young people to transition healthily into adulthood: (1) Competence, involving a positive view of one’s actions in specific areas such as social, academic, cognitive, and vocational; (2) Confidence, reflecting an internal sense of positive self-esteem and general self-efficacy; (3) Connection, establishing positive bonds with key individuals and institutions for their development (family, peers, school, and community); (4) Character, involving respect for and adherence to sociocultural norms, as well as an ethical and moral sense of right and wrong; and (5) Caring/Compassion, understood as a sense of sympathy and empathy towards others [1, 24, 26]. When these 5Cs are present, young people are less likely to engage in risky behaviors, experience fewer mental health problems, and ultimately develop a “sixth C”, characterized by positive contributions to their immediate context and, ultimately, society [27, 31].

Over the last decade, research has shown significant relationships between the 5Cs of PYD and constructs relevant to adolescent well-being and mental health. For instance, in a study involving over 7,000 North American adolescents, Geldhof et al. [32]. observed that both positive youth development (PYD) and the 5Cs correlated with relevant criterion measures, such as depressive symptoms and behavioral problems. Consistently, a study in Ireland found that PYD was related to decreased depressive symptoms and risky behaviors, while also being associated with greater contribution [33]. In Norway, researchers found that the 5Cs were positively correlated with life satisfaction and empowerment and negatively correlated with depressive and anxiety symptoms [22]. On the other hand, Kozina et al. [34]. conducted a study with Portuguese, Slovenian, and Spanish adolescents, finding significant associations between the 5Cs of PYD and anxiety. More recently, Novak et al. [35]. conducted a study with Croatian adolescents, reporting positive associations between the 5Cs and mental well-being, as well as negative associations with emotional/mental distress. The latter is understood as a measure of vulnerability to different mental disorders, including depression, anxiety, and stress [36, 37].

In general, the results suggest that the 5Cs of positive youth development contribute to a lower risk of experiencing mental health problems among adolescents. However, it is important to note that most of these findings come from samples that can be considered WEIRD (an acronym for ‘Western, Educated, Industrialized, Rich, and Democratic’), meaning they are primarily composed of individuals from Western, highly educated, industrialized, wealthy, and democratic societies. This raises concerns about the generalizability of these results to more diverse populations [38].

While research on the Lerner model in non-Western contexts is on the rise [39–41], empirical evidence concerning the 5Cs of Positive Youth Development within Latin American settings remains scarce. Only recently have a few studies begun to explore Lerner’s proposition of the 5Cs. For example, Tirrell et al. [42] provided evidence of their relationship with spirituality and hope among Salvadorian youth, Domínguez Espinosa et al. [43] linked them to healthy lifestyle behaviors in Mexican adolescents, and Manrique-Millones et al. [44] associated them with depressive symptoms among university students in Peru. Furthermore, an expanded version of the model, the 7Cs, was examined by Manrique-Millones et al. [45], which found consistent correlations with risky behaviors such as alcohol and drug use, violence, and suicide attempts among youth in Colombia and Peru. Regarding the Chilean context, the study of 5Cs of PYD initiated with the linguistic adaptation of the PYD-Scale Short Form by Marín-Gutiérrez et al. [46], which provided initial evidence of validity, internal consistency, and significant links with self-esteem, anxiety, and depression; however, there are no further studies.

Despite these initial findings, none of the previous studies have investigated the specific behaviors of each of the Cs of PYD in relation to various specific and general indicators of emotional distress. Moreover, given the cultural diversity across Latin American countries, it is expected that young people will exhibit differences in their personal experiences, worldviews, resources, and opportunities [25, 28]. Therefore, it remains pertinent to continue questioning whether the 5Cs model and the positive outcomes derived from its theoretical foundation also apply to Chilean adolescents.

In this regard, the present study aims to contribute to fill the existing gap in the scientific literature by understanding the mechanisms that promote mental health among adolescents. Specifically, it explores the relationship between the 5Cs of Positive Youth Development and four indicators of mental health problems: depression, anxiety, stress, and emotional distress, in a sample of Chilean adolescents. The central research question is: How are the 5Cs of Positive Youth Development related to various indicators of emotional distress in Chilean adolescents? To address this question, the following hypotheses are proposed:

- H1: The 5Cs of PYD are negatively associated with depression.
- H2: The 5Cs of PYD are negatively associated with anxiety.
- H3: The 5Cs of PYD are negatively associated with stress.
- H4: The 5Cs of PYD are negatively associated with emotional distress.

## Method

### Study design

The study corresponds to a quantitative, non-experimental, cross-sectional research design with correlational-explanatory scope. The subjects of the study were adolescents enrolled in secondary education in the Arica and Parinacota and Tarapacá regions in Northern Chile. The sampling method was non-probabilistic and purposive, implying that participants were selected based on their accessibility.

### Participants

The sample consisted of 425 adolescents, all of whom were students from subsidized private schools^1^ in three cities in Northern Chile: 97 from Arica (23%), 134 from Alto Hospicio (32%), and 194 from Iquique (46%). The ages of the participants ranged from 12 to 19 years (M = 14.95; SD = 1.81). Regarding their gender, 172 identified as male (40.5%), 238 as female (56%), 6 as transgender male (1.4%), one as transgender female (0.2%), and 8 as non-binary (1.9%). In terms of family structure, 217 (51.3%) came from two-parent families, 184 (43.5%) from single-parent families, and 22 (5.2%) from other family arrangements (adolescents living with relatives other than their parents). Regarding ethnicity, 333 (78.7%) reported no ethnic affiliation, while 90 (21.3%) identified with an ethnic group, specifically 56 (13.2%) as Aymara, 14 (3.3%) as Mapuche, 10 (2.4%) as Diaguita, and 9 (2.1%) from other Indigenous groups. The nationalities of the participants were as follows: 376 (88.9%) Chilean, 17 (4.0%) Bolivian, 13 (3.1%) Peruvian, 7 (1.7%) Venezuelan, and 10 (2.4%) from other countries.

### Variables and instruments

#### 5Cs of positive youth development

The Positive Youth Development Scale - Short Form (PYD-SF) [47], adapted into Spanish for Chilean adolescents [46] was used to assess the 5Cs of Lerner’s positive youth development model [1, 24]: Competence, Confidence, Connection, Character, and Caring/Compassion. The instrument uses a five-point Likert-type response format ranging from 1 (Strongly Disagree) to 5 (Strongly Agree). It is worth noting that the adapted version of the PYD-SF consists of 33 items instead of the original 34-item scale, as item 5 (“I hardly ever do things I know I shouldn’t do”) was discarded due to the absence of significant factorial saturation with the hypothesized dimension [39]. However, to adhere to best measurement practices, its content was readapted (“I avoid doing things I know are wrong”) to keep its original structure of 34 items. The psychometric properties of the 34-item version of the PYD-SF for this sample were satisfactory and are reported in the results section.

#### Depression, anxiety, and stress

To assess indicators of emotional distress, the Depression, Anxiety, and Stress Scales (DASS-21) [36], adapted for use in Chilean adolescents [48], were used. The DASS-21 is an instrument that evaluates the presence of symptoms of depression, anxiety, and stress from a dimensional perspective of these psychological disorders. It features four response alternatives in Likert format, ranging from 0 (Did not apply to me at all) to 3 (Applied to me very much, or most of the time). Each scale consists of seven items, and the total score is obtained by summing the items corresponding to that scale, with a score range from 0 to 21 points. Regarding its internal consistency, the coefficients obtained for the present sample are reported in the results section.

### Procedure

This research received approval from the Scientific Ethics Committee of the Universidad de Tarapacá (CEC-UTA). Contact was established with educational institutions, and technical meetings were held with school principals and teachers from the participating schools. After coordinating available dates for the study, the following was obtained: (a) authorization through informed consent from parents, guardians, and tutors; (b) informed assent from those students who chose to participate voluntarily. The purpose of the study, procedures, and participants’ rights were explained to guardians in parent meetings and to students before the questionnaires were administered. Data collection took place within the school premises, in sessions with an average duration of 30 min, using paper-and-pencil format, conducted collectively and assisted, always supervised by at least one of the researchers. Finally, questionnaire responses were transferred to electronic format using a spreadsheet to build the database for analysis.

### Data analysis

**Fig. 1:**
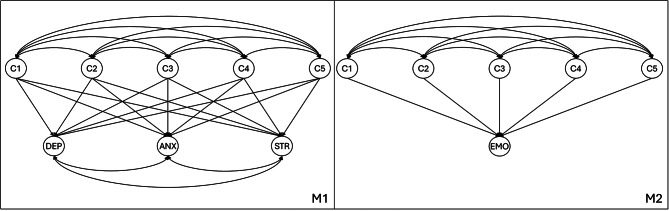
Analyzed Structural Equation Models (SEM) of the 5Cs of Positive Youth Development on Criterion Variables. *Note*: Observed variables have been omitted to facilitate the schematic representation of structural equation models (SEM). M1 represents the hypothesized relationship of the 5Cs of PYD on depression (DEP), anxiety (ANX), and stress (STR). M2 represents the hypothesized relationship of the 5Cs of PYD on emotional distress (EMO)

Preliminary statistical analyses were conducted using the Jamovi 2.5 statistical package [49] to describe data central tendency, dispersion, and distribution shape, and to assess univariate normality using the Shapiro-Wilk test (W). The internal consistency reliability of the scales was examined by calculating Cronbach’s alpha (α) and McDonald’s omega (ω) coefficients, with values above 0.70 considered acceptable. Subsequently, a series of confirmatory factor analyses (CFA) and structural equation models (SEM) were conducted using Mplus software v.8.2 [50]. To evaluate model adequacy for the study sample, a model with five correlated factors and a refined version including observed variables from the PYD-SF [46] were estimated. For the DASS-21 indicators, two models were estimated: a three-correlated factors model previously reported in a study with Chilean secondary school students [48] and a bifactor model with a general factor named “emotional distress” and three specific factors [37]. Two structural equation models (SEM) were then performed to test study hypotheses, one for depression, anxiety, and stress, and the other for emotional distress (see Fig. 1), using the five dimensions of the PYD-SF as independent variables. Both CFA and SEM used the Weighted Least Squares Mean and Variance (WLSMV) estimation method [51]. Model fit was assessed using multiple indicators: a ratio of χ²/df < 5, RMSEA < 0.08, CFI and TLI > 0.90 indicated acceptable fit, while a ratio of χ²/df < 2, RMSEA < 0.06, CFI and TLI > 0.95 indicated excellent fit [52, 53] Additionally, a SRMR < 0.08 was considered indicative of acceptable fit between the hypothesized model and the observed data [54].

## Results

### Preliminary analysis

Table 1 presents descriptive statistics, univariate normality, and alpha and omega coefficients for each dimension of the PYD-SF and the DASS-21. The proportion of valid cases exceeded 96.9% for all variables under analysis. Shapiro-Wilk test results were significant, indicating that the variables did not follow a normal distribution. Additionally, alpha and omega coefficients were above 0.70, indicating good internal consistency across all evaluated dimensions.

**Table 1:** Descriptive statistics, normality, and internal consistency of PYD-SF and DASS-21 dimensions

Dimension	*N*	M	SD	Skew	Kurt	W	α	ω
Competence	423	18.96	4.65	-0.11	-0.39	0.99**	0.73	0.74
Character	417	29.84	5.08	-0.59	0.59	0.98***	0.74	0.75
Confidence	420	19.48	6.24	-0.21	-0.81	0.97***	0.90	0.90
Caring	418	23.43	4.76	-0.77	0.38	0.94***	0.85	0.86
Connection	421	27.43	6.05	-0.20	-0.34	0.99**	0.81	0.81
Depression	420	9.25	6.37	0.25	-1.10	0.95***	0.91	0.91
Anxiety	418	8.96	6.05	0.38	-0.96	0.95***	0.87	0.87
Stress	418	9.89	6.05	0.14	-1.07	0.96***	0.89	0.89
Emotional Distress	412	28.19	17.13	0.23	-1.05	0.96***	0.95	0.95

Note: *p < 0.05, **p < 0.01, *p* < 0.001***

### Measurement models

Goodness-of-fit indices for the measurement models of the PYD-SF and DASS-21 are presented in Table 2. Regarding the PYD-SF, the correlated five-factor model (5 F in Table 2) exhibited unsatisfactory fit (χ2/df = 3.887, RMSEA = 0.084 (90% CI = 0.080 – 0.088), CFI = 0.891, TLI = 0.882, and SRMR = 0.081). However, guided by the analyses of Marín-Gutiérrez et al. [46] and modification indices proposed by the statistical report, we proceeded to examine a refined version of the model. The re-specification of the correlated five-factor model of the PYD-SF (5 F* in Table 2) adequately fit the data (χ2/df = 3.049, RMSEA = 0.071 (90% CI = 0.067 – 0.075), CFI = 0.923, TLI = 0.916, and SRMR = 0.074), showing a significant improvement compared to its predecessor model (χ2Δ = 287.493, df = 3, *p* < .001). Item factor loadings were significant for each factor (*p* < .001) and ranged from 0.438 to 0.916. Meanwhile, correlations between factors were significant (*p* < .001) and direct (competence × character = 0.578; competence × confidence = 0.783; competence × compassion = 0.195; competence × connection = 0.814; character × confidence = 0.481; character × compassion = 0.780; character × connection = 0.654; confidence × connection = 0.763; compassion × connection = 0.369). The lowest correlation was observed between the confidence and compassion factors (*r* = .093, *p* = .049). Regarding the DASS-21, two measurement models were evaluated. Firstly, the theoretical model of three correlated factors (3 F in Table 2) was tested, which exhibited satisfactory fit (χ2/df = 3.066, RMSEA = 0.071 [90% CI = 0.064 – 0.077], CFI = 0.977, TLI = 0.974, and SRMR = 0.038). The factor loadings for this measurement model were significant (*p* < .001) and ranged between 0.487 and 0.923. The factors obtained significant (*p* < .001) and high-magnitude direct correlations (depression × anxiety = 0.856; depression × stress = 0.844; anxiety × stress = 0.943). Secondly, the fit of a bifactor model (BF in Table 2) was explored, characterized by a general factor of emotional distress and three specific factors. This latter model demonstrated an excellent fit to the data (χ2/df = 1.991, RMSEA = 0.049 [90% CI = 0.041 – 0.057], CFI = 0.990, TLI = 0.988, and SRMR = 0.027). The factor loadings of emotional distress were significant in all its items (*p* < .001) and ranged from 0.484 to 0.867. The factor saturation of the specific factors was lower (FL > 0.103 and < 0.724) and not significant in five of its elements: item 2, 7, and 9 for anxiety, and item 8 and 18 for stress.

**Table 2:** Global fit indices of measurement models

Model	χ2	Gl	*p*	χ2/gl	CFI	TLI	RMSEA [IC 90%]	SRMR
PYD-SF								
5 F	2009.711	517	0.000	3.887	0.891	0.882	0.084 [0.080 – 0.088]	0.081
5 F*	1567.536	514	0.000	3.049	0.923	0.916	0.071 [0.067 – 0.075]	0.074
χ2Δ 5 F vs. 5 F*	287.493	3	0.000					
DASS-21								
3 F	570.340	186	0.000	3.066	0.977	0.974	0.071 [0.064 – 0.077]	0.038
BF	328.607	165	0.000	1.991	0.990	0.988	0.049 [0.041 – 0.057]	0.027

Note * Respecified. Model 5 F* (PYD-SF) includes the covariation of errors of three pairs of items: 3–19, 14–31, and 17–34

### Structural equation models (SEM)

The 5Cs of PYD and the indicators of mental health problems (depression, anxiety, stress, and emotional distress) are represented as latent variables in their respective structural models (see Fig. 1). Each latent variable is estimated from its respective indicators: (a) the 5Cs of PYD are estimated with 34 observed variables; (b) the specific dimensions of the DASS-21 with seven variables each; (c) the general factor of the DASS-21 with 21 variables. The overall fit of the SEMs is presented in Table 3. Both models, M1 and M2, showed satisfactory fit to the data. M1, which represents the relationship between the 5Cs of PYD (competence, character, confidence, compassion, and connection) on the three specific dimensions of the DASS-21, exhibited good fit, with indicators such as CFI and TLI surpassing the threshold of 0.95, and an RMSEA within acceptable limits (χ2 = 2502.803, df = 1399, *p* < .001, χ2/df = 1.788, CFI = 0.957, TLI = 0.954, RMSEA = 0.044 [90% CI: 0.041–0.047], SRMR = 0.061). This model explained a significant change in the variance of depression symptoms (43.1%, *p* < .001), anxiety (36.5%, *p* < .001), and stress (31.3%, *p* < .001). Similarly, M2, which explores the relationship between the dimensions of 5 C of PYD and the general factor of the DASS-21, also showed a solid fit to the data (χ2 = 2469.694, df = 1398, *p* < .001, χ2/df = 1.766, CFI = 0.958, TLI = 0.955, RMSEA = 0.044 [90% CI: 0.041–0.046], SRMR = 0.061), explaining 39.4% of the variance in emotional distress (*p* < .001).

**Table 3:** Overall fit indices of structural models

Model	χ2	gl	*p*	χ2/gl	CFI	TLI	RMSEA [IC 90%]	SRMR
M1	2502.803	1399	0.000	1.788	0.957	0.954	0.044 [0.041 – 0.047]	0.061
M2	2692.169	1412	0.000	1.906	0.950	0.947	0.047 [0.045 – 0.050]	0.065

Note M1 = Respecified five-factor model of 5Cs of positive youth development on depression, anxiety, and stress; M2 = Respecified five-factor model of 5Cs of positive youth development on emotional distress

Table 4 displays the standardized effects of the 5Cs of PYD on the dependent latent variables of both structural models (M1 and M2). Examination of the observed effects revealed that confidence has a significant direct effect on depression (-0.498, *p* < .001), anxiety (-0.342, *p* < .001), stress (-0.263, *p* < .001), and emotional distress (-0.406, *p* < .001). Similarly, connection has a significant direct effect, although somewhat weaker, on depression (-0.241, *p* < .05), anxiety (-0.309, *p* < .05), stress (-0.316, *p* < .05), and emotional distress (-0.292, *p* < .01). In M1, caring also stands out for its direct and positive effect on stress symptoms (0.376, *p* < .05).

**Table 4:** Standardized effects of the 5Cs of PYD on depression, anxiety, stress, and emotional distress

	M1			M2
	DEP	ANX	STR	EMO
Competence	− 0.038	− 0.146	0.087	− 0.026
Character	0.153	0.183	− 0.221	0.087
Confidence	− 0.498***	− 0.342***	− 0.263**	− 0.406***
Caring	0.055	0.027	0.376*	0.151
Connection	− 0.241*	− 0.309*	− 0.316*	− 0.292**

Note DEP = Depression; ANX = Anxiety; STR = Stress; EMO = Emotional distress; *p < 0.05, **p < 0.01, *p* < 0.001***

## Discussion

The purpose of this study was to investigate the impact of the 5Cs of positive youth development (PYD) on depression, anxiety, stress, and emotional distress among Chilean adolescents. The results suggest that only two out of the 5Cs of PYD demonstrate significant associations with the four indicators of mental health problems examined.

The evaluation of the dimensionality of the Positive Youth Development-Short Form Scale (PYD-SF) followed the recommendation of Marín-Gutiérrez et al. [46], which involved the readaptation of the content of item 5 before subjecting it to confirmatory factor analysis (CFA). Following the CFA, examination of modification indices suggested a re-specification of the model, involving the addition of parameters such as the covariation of errors between three pairs of items: (a) items 3 and 19 (Confidence), referring to perceptions of physical and/or athletic self-efficacy; (b) items 14 and 31 (Connection), related to support provided by the school environment; and (c) items 17 and 34 (Connection), concerning positive peer relationships. These adjustments to the model were consistent with those previously reported by Tomé et al. [55] with Portuguese adolescents, and Marín-Gutiérrez et al. [46] with Chilean adolescents. Moreover, subsequent analysis of the internal consistency of the instrument proved to be satisfactory, yielding alpha and omega coefficients equal to or greater than 0.73. Taken together, these findings provide evidence regarding the utility of the PYD-SF in consistently evaluating the 5Cs model of positive development in samples of Chilean adolescents.

The factorial analysis of the DASS-21 satisfactorily replicated the theoretical three-factor structure (depression, anxiety, and stress), as also evidenced by Mella et al. [48] in a sample of high school students from Southern Chile. As an innovation to the study of the internal validity of the DASS-21 in Chilean adolescent population, a bifactor model with a general factor and three specific factors proposed by Valencia [37] in his study with university students from Peru was examined. In this regard, the general factor, termed Emotional Distress (EMO), is operationalized as a latent factor in which the scores of the 21 items of the instrument saturate. The fit of the bifactor model was excellent and suggests that the dimensions of the DASS-21 can be interpreted both as three specific dimensions (depression, anxiety, and stress) and as a global dimension of EMO. In this sense, EMO can be understood as a measure of vulnerability to different mental health problems characterized by negative affective states [35, 37].

The structural models provided evidence that partially supports the hypotheses regarding the relationship between the 5Cs of PYD and the four indicators of mental health problems included in the study. Specifically, models M1 and M2 demonstrated that two of the 5Cs (confidence and connection) exert a direct negative effect on depression, anxiety, stress, and emotional distress. This implies that adolescents who report high levels of self-confidence and maintain positive and stable bonds with key individuals and institutions in their context are less likely to experience symptoms of emotional distress. The effect of confidence and connection on these indicators aligns with recent research conducted by Novak et al. [35], where they examined the discriminant validity of the 5Cs model in Croatia. In this study, the depression, anxiety, and stress scales of the DASS-21 were used as criterion variables, along with a latent variable reflected through the total scores of each scale. However, unlike this research, the present study did not demonstrate the expected protective effect of the remaining 5Cs: competence, character, and caring.

Unexpectedly, it was identified that caring has a positive direct effect on symptoms of stress in adolescents, suggesting that young people who demonstrate compassion towards others’ problems may experience tension, irritability, and fatigue as a direct consequence of their caregiving efforts. Although this study does not address the role of gender in the expression of compassion by adolescents, reflection on traditional gender roles offers a possible interpretation of these results. Historically, women have more frequently assumed caregiving roles, thus facing a significant emotional and physical burden. This raises the question of whether the internalization of such gender roles could be exacerbating stress experiences among adolescents actively engaged in caregiving tasks, perhaps due to pressure to adhere to social norms and the emotional demands associated with caregiving. However, such considerations will need to be explored in future research.

These results suggest variability in the contribution of each dimension of PYD to adolescents’ mental health, which could be explained by differences of various kinds. These include cultural aspects, such as expectations, values, and sociocultural norms associated with youth; socioeconomic factors, such as access to resources and opportunities that shape adolescents’ developmental trajectories and, consequently, their mental health; the school system and the educational project of educational institutions, which could affect how PYD dimensions are promoted and experienced; and environmental elements, such as geography, climate, and community relationship structures.

Supporting these explanations, recent international studies compiled in the Positive Youth Development Handbook by Dimitrova and Wiium [38], suggest that young people around the world differ in how the 5Cs are configured and expressed, and that these differences are shaped by the sociocultural context in which they are immersed. However, in the Latin American context, it is necessary to continue gathering more data to begin questioning whether theoretical models of positive youth development should be proposed that are more rooted in the cultural realities of adolescents or to continue searching for models that establish general regularities in this population.

In relation to the situation in Chile, where the 5Cs model of positive development has received little attention from researchers, the findings of this study provide support for the proposal by Lerner [1, 24]. The identification of the specific role that each of the 5Cs plays in mental health problems suggests that psychosocial interventions aimed at reducing adolescents’ vulnerability to experiencing symptoms of depression, anxiety, stress, and emotional distress should prioritize strengthening both adolescents’ self-confidence and the positive connections they maintain with others. This underscores the need to adopt a comprehensive approach to addressing adolescents’ mental health [2].

Integrating the principles of the 5Cs into both school and community mental health programs would enable more effective outcomes in protecting mental health and enhancing adolescent development. This approach includes strategies that strengthen the dimensions of Confidence and Connection by: (1) establishing systems that recognize and celebrate adolescents’ achievements and efforts, not only in academics but also in extracurricular and personal activities; (2) conducting workshops on self-awareness and personal development to help students explore their identity, identify their strengths and areas for improvement, and foster a positive self-view; (3) offering parent workshops that educate about adolescent development, conflict management, and effective communication techniques to improve the quality of parent-child relationships; (4) organizing family activities such as cultural fairs and sports days to promote effective alliances between families and schools; and (5) implementing community service projects that engage students in initiatives benefiting the local community, fostering a sense of responsibility and belonging among youth.

Regarding the strengths of the study, it must be acknowledged that the research adds knowledge to the literature on positive youth development and mental health in adolescents, especially in the Chilean context, where research on the 5Cs has been limited. Additionally, it employs advanced statistical analyses, such as confirmatory factor analysis (CFA) and structural equation modeling (SEM), to evaluate the dimensionality and proposed relationships in the theoretical framework. In line with this, the findings provide contextualized guidance for the development of psychosocial interventions aimed at promoting the mental health of Chilean adolescents.

As for its limitations, it is important to note that its cross-sectional design makes it difficult to draw causal conclusions about the interrelationship between the 5Cs and the examined criterion indicators. Additionally, the use of a convenience sample to obtain these results invites interpreting the findings with some caution regarding their generalizability. Finally, obtaining data on mental health problems through self-reporting may introduce perception biases, as some participants, especially younger ones, may have difficulty accurately assessing their own experiences and behaviors.

Future research could significantly mitigate the limitations presented in this study by accumulating evidence through replicating the research with samples of adolescents from diverse geographical locations in Chile or Latin America. The adoption of longitudinal designs would allow capturing temporal dynamics and offering a deeper understanding of the causal relationships between the 5Cs and indicators of mental health problems. Additionally, to enhance the validity of the data, it is suggested to complement the information on symptoms provided by adolescents by incorporating measures of hetero-report obtained from parents and/or teachers. Moreover, it is desirable to extend the line of research to explore the role played by the 5Cs in relation to other mental health problems that were not considered in the present study, including behavioral problems, substance abuse and/or dependence, and eating disorders. Additionally, investigating the influence of the model on other positive outcomes, such as psychological well-being, prosocial behavior, and academic achievement, would allow for a more comprehensive understanding of the influence of the 5Cs on the mental health and overall development of adolescents.

## Data Availability

The data analyzed in this research cannot be shared publicly at this time. This decision is based on the fact that the data in question are a critical part of an ongoing doctoral project led by the corresponding author. Since the research is still in progress, releasing the data at this stage could compromise the integrity and outcomes of the doctoral project.

## Footnotes

1 The Chilean school system is a mixed system that includes public and private schools, the latter of which may or may not receive government subsidies. According to recent data from the Ministry of Education [56], subsidized private schools constitute the most common type of establishment, representing 49.2% of national educational coverage, 63.3% in Arica, 61.8% in Iquique, and 93.6% in Alto Hospicio.
